# Murine Models of Central Nervous System Disease following Congenital Human Cytomegalovirus Infections

**DOI:** 10.3390/pathogens10081062

**Published:** 2021-08-21

**Authors:** Jerome Moulden, Cathy Yea Won Sung, Ilija Brizic, Stipan Jonjic, William Britt

**Affiliations:** 1 Department of Microbiology, UAB School of Medicine, Birmingham, Al 35294, USA; moulden1@uab.edu; 2 Laboratory of Hearing Biology and Therapeutics, NIDCD, NIH, Bethesda, MD 20892, USA; cathy.sung@nih.gov; 3 Center for Proteomics and Department of Histology and Embryology, Faculty of Medicine, University of Rijeka, 51000 Rijeka, Croatia; ilija.brizic@medri.uniri.hr (I.B.); stipan.jonjic@medri.uniri.hr (S.J.); 4 Department of Pediatrics and Neurobiology, UAB School of Medicine, Birmingham, Al 35294, USA

**Keywords:** congenital CMV, CNS viral infection, infection during neurodevelopment

## Abstract

Human cytomegalovirus infection of the developing fetus is a leading cause of neurodevelopmental disorders in infants and children, leading to long-term neurological sequela in a significant number of infected children. Current understanding of the neuropathogenesis of this intrauterine infection is limited because of the complexity of this infection, which includes maternal immunological responses that are overlaid on virus replication in the CNS during neurodevelopment. Furthermore, available data from human cases are observational, and tissues from autopsy studies have been derived from only the most severe infections. Animal models of this human infection are also limited by the strict species specificity of cytomegaloviruses. However, informative models including non-human primates and small animal models have been developed. These include several different murine models of congenital HCMV infection for the study of CMV neuropathogenesis. Although individual murine models do not completely recapitulate all aspects of the human infection, each model has provided significant information that has extended current understanding of the neuropathogenesis of this human infection. This review will compare and contrast different murine models in the context of available information from human studies of CNS disease following congenital HCMV infections.

## 1. Introduction

Perinatal infections caused by human cytomegalovirus (HCMV) are common and include both infections acquired in utero (congenital HCMV) and those acquired in the peripartum period and in early infancy. Although clinically significant perinatal infections occur in premature infants who acquire HCMV early in life, most severe HCMV infections and, in particular, those associated with long-term neurodevelopmental sequelae, result from intrauterine transmission of HCMV to the developing fetus. Congenital HCMV (cCMV) infection is relatively frequent in most regions of the world, with birth prevalence ranging from 5 to 10 cases per 1000 live births [1,2,3]. Thus, HCMV is by far the most frequent intrauterine viral infection in humans. Infants infected in utero can present with clinical evidence of multiorgan disease at birth, an infection which is classified as symptomatic, or alternatively, without clinical symptoms, a presentation described as asymptomatic infection. Findings in infants with symptomatic infections can include dysfunction in multiple organ systems with disordered hepatic function, hematological abnormalities, growth restriction, and perhaps most importantly for long-term outcomes, evidence of CNS damage [4,5] (Table 1). The clinical findings in infants with CNS involvement can include microcephaly with significant structural brain damage, retinitis, hearing loss, and vestibular dysfunction (Table 1). Neurodevelopmental sequelae are more commonly described in infected infants with symptomatic cCMV infections and who exhibit clinical manifestations of CNS involvement [6]. Importantly, a subset of infants with asymptomatic cCMV infection without clinical evidence of CNS infection can also exhibit neurodevelopmental abnormalities, including permanent hearing loss and vestibular dysfunction (Table 1) [7,8]. Intrauterine infection acquired early in gestation is a risk factor that has been consistently associated with significant CNS disease in cCMV-infected infants, a finding consistent with the impact of HCMV infection of the CNS during very early periods of neurogenesis in the brain [9,10,11,12]. Due to the contribution of CNS infection to adverse long-term outcomes in infants with cCMV infections, considerable effort has been directed towards defining mechanisms of CNS infection and disease that follow intrauterine HCMV infections. In humans, the vast majority of these studies are observational and have attempted to correlate or associate outcomes of infected infants with clinical and laboratory findings. Efforts to more accurately define mechanisms of CNS disease associated with cCMV infection have relied on small animal models and, less frequently, non-human primate models. Each of these models employs their respective CMV because HCMV and most other mammalian CMVs are exquisitely species-specific. In the case of HCMV, replication with the production of infectious progeny virus takes place only in human cells and cells from some non-human primates. Although there are obvious limitations in each of these animal models, data generated from studies in each of these model systems when coupled with the more limited amount of data derived from studies in human tissues have produced considerable insight into mechanisms of CNS disease resulting from HCMV infection of the developing CNS. Translation of these findings into interventions that could limit sequelae of cCMV infections now seems within reach. This review will focus on murine models that have been developed to investigate the mechanisms of central nervous system (CNS) damage associated with HCMV infection of the developing CNS in infants infected in utero.

## 2. Central Nervous System Disease following Congenital HCMV Infections

As shown in Table 1, clinical findings of CNS involvement are present in approximately 50% of infants with clinically apparent or symptomatic cCMV infections, and in about 10% of infected infants who present without clinical findings [6,13]. Early studies of autopsy tissue obtained from infected stillborn infants and infants who died in the perinatal period with cCMV infections relied on the detection of characteristic histopathological findings of enlarged cells with intracellular and intranuclear inclusions, the so-called “owls eyes” inclusions, characteristic of cells productively infected with HCMV. These studies described a number of findings that included gross pathological changes associated with severe structural brain damage, such as lissencephaly, polymicrogyria, cerebellar hypoplasia, and ventriculomegaly [14]. In most cases, these findings were associated with a loss of brain parenchyma and were consistent with the clinical findings of microcephaly in infected infants. Importantly, in almost all cases, these changes were symmetric and not associated with focal areas of tissue necrosis. This gross pathologic characteristic of CNS damage in cCMV infections contrasts with the asymmetric nature of the necrotizing encephalitis that is commonly described in CNS tissues from newborn infants infected with the alphaherpesvirus, herpes simplex virus (HSV). In contrast to these findings in infants with symptomatic infections, gross pathological changes in the CNS such as those described above have not been reported in infants with subclinical cCMV infections who died in the perinatal period from other disease processes unrelated to cCMV infections. However, it is important to note that evidence of CNS involvement has been described in infected infants without clinical findings of cCMV infections. The findings from a large autopsy series carried out in Auckland, New Zealand, which included 90% of all perinatal deaths from 1964 to 1979, provided evidence of HCMV CNS infections in infants that were liveborn but without findings of cCMV infection and subsequently expired from other causes [14]. Of the nine infants that were subsequently identified as having cCMV infection based on typical HCMV cytopathology in autopsy tissue four (4/9, 45%) had findings described as acute encephalitis, with the most common finding being focal encephalitis with mononuclear cell infiltrates [14]. Although this study was clearly limited by the reliance on histopathology to identify HCMV-infected infants, it nonetheless demonstrated that even in infected infants without clinical findings consistent with cCMV infections, CNS involvement can occur and thus may contribute to long-term neurological sequelae that have been documented in infants with asymptomatic cCMV infections. It is also of interest that the pathological lesions described in this report as acute focal encephalitis closely resembled the predominant type of histopathological lesions in the brains of immunocompromised hosts that have been described as microglial nodule encephalitis, or alternatively, as micronodular gliosis [15].

More recently, the histopathology of HCMV infections in the CNS of developing fetuses has been more comprehensively described in studies of tissue from aborted fetuses derived from terminated pregnancies. In the majority of these cases, prenatal imaging and diagnostic testing were used to identify these infected fetuses, and in some cases, imaging abnormalities were identified in the developing fetal brain. These studies have used contemporary technologies together with conventional histopathology to provide detailed descriptions of the characteristics of HCMV infection in the developing CNS. In two large autopsy series which included fetal tissue obtained between 20 and 28 weeks of gestation, lesions in the brain were identified in the majority of fetuses [16,17]. In both studies, clinical findings of the maternal HCMV infection and the pathologic findings in the infected brains were most consistent with intrauterine HCMV transmission during the first trimester (Table 2) [16,17]. Evidence of specific neural cell tropism was not observed in either study, and HCMV could be detected by immunohistochemistry or histopathology in neurons, glia, and in one report, endothelial cells [16,17]. Similar to findings that have been described in studies of immunocompromised adults with HCMV encephalitis, the predominant histopathologic finding was described as micronodular panencephalitis in which focal micronodular lesions consisting of mononuclear cells surrounding HCMV-infected cells were observed in most regions of the brain, including the olfactory bulb in about one-third of fetuses described by Teissier et al. [15,16,17]. The density of these histopathological lesions in different regions of the brain was shown to correlate with the viral load, as quantified by PCR [17]. In addition, infection of neural progenitors in the subventricular zone (SVZ) was correlated with ventricular dilation, cortical dysplasia, loss of cortical parenchyma, and microcephaly, suggesting that a loss of neural stem/progenitor cells in these regions of the developing brain contributed to pathologic changes in the brains of these cases [16]. In both of these autopsy studies, a robust infiltrate of Iba1+ mononuclear cells and CD8+ lymphocytes was observed, suggesting a fetal response to tissue infection with HCMV [16,17]. More recently, a study of 26 fetal brains from HCMV-infected fetuses obtained following the termination of pregnancy secondary to fetal ultrasound abnormalities reported infiltrations of CD8^+^ and CD20^+^ lymphocytes and CD68^+^ macrophages in periventricular areas of the brain [18]. In more severely affected brains, NK cells were detected by immunostaining with NKp46 and NKG2C antibodies [18]. The magnitude of cell infiltration of the adaptive and innate response was correlated with the magnitude of viral infection, as detected by the density of HCMV-infected cells, a finding consistent with a fetal response to this viral infection of the brain [18]. Other smaller studies have reported findings consistent with these large series, and together, results from the available studies suggest several potential mechanisms of disease, including: (i) direct viral cytopathology leading to a loss of neural stem/progenitor cells, resulting in the loss of brain parenchyma; (ii) deficits in proliferation and/or migration of neural progenitor cells induced by virus infection, including paracrine effects; and (iii) indirect virus-induced neuropathology secondary to host inflammatory responses.

**Figure 1:**
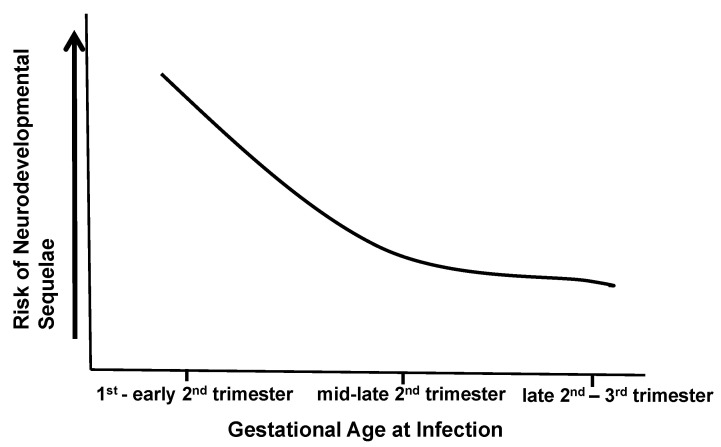
Adverse outcomes of congenital CMV infections represent a continuum. The risk of long-term neurodevelopmental sequelae following intrauterine CMV infection cannot be represented as a binary risk, i.e., symptomatic or asymptomatic infection, but reflects the severity of CNS damage. Infections later in pregnancy are associated with fewer long-term neurological sequelae than infections that occur early in pregnancy, during periods of neurogenesis. Similarly, direct virus-induced cytopathology could represent a major component of the neuropathogenesis of infections early in pregnancy, whereas disease following intrauterine infections occurring later in pregnancy could be secondary to indirect mechanisms, including immunopathological responses. Other parameters of fetal infection, including viral load, maternal immune responses, and perhaps fetal immune responses, could modify long-term outcomes of fetal infection.

A large number of HCMV-infected fetuses and newborn infants have been studied with different neuroimaging methodologies. Early studies in infants with symptomatic infections revealed a variety of abnormal findings, regardless of whether computerized tomography (CT) or magnetic resonance imaging (MRI) were employed. In early studies that used only CT to study infants with symptomatic cCMV infections, periventricular and cerebral calcifications were the most common abnormality with ventriculomegaly and cortical atrophy, and in some cases, encephalomalacia was also described [19,20,21]. Not surprisingly, the severity of CNS imaging abnormalities was correlated with the severity of neurological abnormalities and the likelihood of long-term sequelae in these infected infants [21]. Over the last two decades, a large number of case series of infants with cCMV infection studied by MRI has been reported. Findings from these imaging studies include lissencephaly, polymicrogyria, ventriculomegaly, cerebellar hypoplasia, and calcifications (Table 2). Early MRI studies of the CNSs of infants with symptomatic cCMV infections provided evidence of cortical migration deficits and suggested a mechanism for cortical abnormalities such as lissencephaly, polymicrogyria and cortical dysplasia observed in infected infants [19,22]. These deficits in cortical development are most consistent with the HCMV infection of neural progenitor cells, and as previously noted, altered proliferation and/or the migration of neural progenitors. The impact of HCMV infection on neural progenitor cells could be secondary either to virus infection or, potentially, to paracrine effects associated with HCMV transcription/replication [23,24,25,26,27,28]. Although the impacts of virus infection on neural progenitor cell proliferation, differentiation, and migration have been described, several reports have outlined the impact of virus infection on the expression of cellular genes that have also been associated with the decreased neurogenesis, differentiation and migration of neural stem cells, including the finding that HCMV infection increased expression of the transcription factor peroxisome proliferator-activated receptor gamma (PPARγ) in neighboring but uninfected cells [25,26]. Together, this body of literature strongly argues that HCMV infection of neural progenitor cells during active neurogenesis in the developing brain can lead to abnormalities, such as lissencephaly, polymicrogyria and cortical dysplasia, that have been reported in fetuses and infants infected in utero with HCMV. Thus, the spectrum of findings in infants with CNS infection associated with cCMV infection undoubtedly reflects variations in the timing of the intrauterine transmission of HCMV, such that fetal infection early in gestation results in more severe structural damage to the developing brain. In addition, it is also highly likely that less well-understood parameters of this intrauterine infection, such as the magnitude and duration of the fetal viremia, qualitative and quantitative characteristics of the maternal adaptive response to HCMV, the nature of the fetal innate and adaptive response to the infection, and perhaps even the genetic composition of the infecting virus(es) could also impact brain development in infected infants. Although findings from neuroimaging studies of infants with cCMV infections have provided insight into specific mechanisms of CNS disease that follow cCMV infections, these studies have also further emphasized the complexity of this infection. However, a more realistic interpretation of these data is that variability in the nature of the maternal infections that result in transmission across a fetal maternal interface (placenta), and similarly, variations in the timing and level of infection of the fetus during both neurodevelopment and immunological development, lead to considerable heterogeneity in the phenotypes of disease in infants with cCMV. The complexity of this infection is perhaps best illustrated by a recent report describing different phenotypes of monozygotic twins with cCMV infections of which most were monochorionic [29]. Furthermore, recent findings from a transcriptomic study of infants with symptomatic and asymptomatic cCMV infections failed to define a pattern of gene expression that could distinguish these two clinical phenotypes, although it should be noted that this study cataloged transcriptomic patterns in peripheral blood cells and not in cells from target tissues such as the CNS [30]. Together, these data suggest that the CNS sequelae associated with cCMV infection likely represent a continuum and cannot be represented by a simple binary clinical classification of symptomatic and asymptomatic cCMV infection. This continuum is perhaps best illustrated by findings from studies that have stratified infants with symptomatic cCMV infections into those with and without CNS involvement, and shown that long-term outcomes are best predicted by CNSinvolvement and not by the presence of a symptomatic infection which can also be defined by clinical findings in peripheral organ systems [31] (Figure 1). Thus, long-term outcomes following cCMV infections are more likely to be defined by the characteristics of CNS infection in the individual infant (Figure 1). Dissecting different pathways that define even a limited number of disease mechanisms in this continuum will be challenging and requires informative in vivo and in vitro models. 

**Table 2:** CNS histopathologic and neuroimaging findings in infants with congenital CMV infections ^1^.

Histopathologic Findings	Imaging Findings
focal microgliosis/nodular gliosis [14,16,17]	calcifications [19,21,32,33,34,35]
peri-ependymitis/peri-ventricular calcifications [14]; ependymitis with CD8+ T lymphocyte infiltrates [17]	disordered migration (lissencephaly; pachygyria, polymicrogyria) [19,22,34,35,36,37]
microglia activation, CD8+ T lymphocyte infiltrates [16,17,18]	cerebellar hypoplasia [22,33,34,36,37]
infection of neural stem cells, astroglial cells, macrophage/microglia [16,17]; endothelial cells [17]	ventriculomegaly [33,34]

^1^ Characteristic laboratory findings in infants with congenital CMV infections.

## 3. Murine Models of CNS Infections Associated with Congenital HCMV Infection: CNS Development in Humans and Rodents

**Figure 2:**
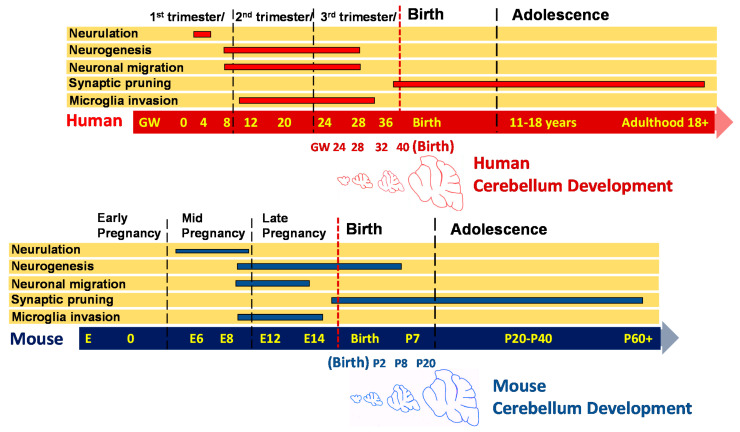
Comparison of human and mouse neurodevelopment. Brain development in both species begins with neurulation and formation of the neural tube. As shown, neurulation begins in the first trimester in humans but later in embryonic development—mid-pregnancy—in mice. Neurogenesis and migration neurons also differ temporally in humans and mice. Yolk-sack-derived microglia invade the CNS and become the resident immune cell of the brain early in development in both species and contribute to synaptic pruning before birth and throughout life. At birth, mouse cerebellar development is comparable to that of a late second trimester human fetus.

As discussed in the previous sections, congenital HCMV infection may be associated with a number of different pathological changes in the developing brain. These virus-induced changes in the brain can range from a severe loss of parenchyma, consistent with defects in the neurogenesis secondary to loss or dysfunction of neural stem cells within the subventricular zones of the developing brain, to minimal gross pathological abnormalities but with histopathological findings that can include inflammatory foci consisting of activated resident and infiltrating mononuclear cells. Although murine models of congenital HCMV infection have been employed to study the neuropathogenesis of congenital HCMV infection, these models are often limited by the lack of reliable transplacental transmissions of MCMV. For this reason, several different strategies have been utilized to infect the murine CNS at different time periods of neurodevelopment. In order to describe the attributes and limitations of different murine models, we will first provide a very limited description of several stages of CNS development in mice in relation to human brain development. An emphasis will be placed on stages relevant to understanding the application of existing murine models for the study of CNS disease following congenital HCMV infection (Figure 2). In general, CNS development in mice and humans largely follows very similar developmental programs, and it has been suggested that the developmental status of the CNS of a newborn mouse is at a similar stage of neurodevelopment as a mid-late second trimester human fetus [38] (Figure 2). However, there are notable differences between these two species in specific developmental periods and in the duration of different periods of neurodevelopment. The initial steps in the formation of the mammalian CNS are described as neurulation, a process that includes neural tube generation. In the human embryo, neurulation begins within the first 4 weeks post-implantation, which is then followed by extensive neurogenesis. In mice, neurulation begins around embryonic day 4 (E4) and proceeds until E8–9. Neurogenesis in the developing human fetus begins around 8 weeks post-conception and extends until the mid-third trimester of gestation. Similarly, neurogenesis begins in the mouse cortex around the completion of neurulation (E8–9) and ends before adolescence. During these time periods, newly born neurons radially migrate to distinct brain regions such that most recently produced neurons occupy the outermost layers of the neocortex. Superimposed on these processes of neurogenesis and migration, extensive cellular connectivity is established between neurons and in different regions of the brain. This critical step in the maturation of the CNS is developmentally regulated and includes both gliogenesis as well as apoptosis as a mechanism for the elimination of redundancy in neural circuits [39]. Interestingly, a number of neurodevelopmental disorders can be associated with failures to establish or to refine cellular connectivity in the developing brain [40]. Microglia, the resident myeloid cells of the CNS, are derived from yolk sac-myeloid cells that invade the human fetal CNS at about 10 weeks post-conception and at around E10 in mice. In addition to a number of functions that contribute to homeostasis in the brain, including the phagocytosis of cell debris and early responses to injury or infection in the brain, microglia are believed to be essential for the pruning of the large number of synapses that form during development, a process that continues after birth. 

In contrast to the human infant, significant development of the murine CNS occurs after birth. This is best illustrated by the development of the cerebellum, one of the most well-studied regions of the brain, which exhibits extensive postnatal development in mice [41,42,43]. In contrast, the human cerebellum begins development 30 days post-conception, where the size and shape of the cerebellar anlage of humans resemble that of mice on E10.5–17.5 [44]. The formation of the external granular layer (EGL) in the human cerebellum begins around 8 weeks post-conception, with peak granular cell precursor proliferation occurring between 26 and 32 weeks post-conception with the disappearance of the EGL within the first year of life [45]. Importantly, in both mice and humans, maturation of cellular connectivity in the cerebellum continues long after birth; it is not complete in humans until later in childhood. Interestingly, this region of the hindbrain contains the largest number of neurons in the human brain, resulting in an extensive surface area that, in itself, represents an estimated 80% of the surface area of the entire neocortex [46]. In addition, this region of the brain has been shown to have a critical role in both motor and cognitive functions of the brain, and abnormalities in cerebellar structure/function have been linked to both cognitive and neuromuscular disorders. 

The murine cerebellum at birth as noted previously is at a developmental stage that is comparable to that of a late second trimester human fetus (Figure 2). In mice, the cerebellar anlage is formed in the hindbrain at around E8.5, and early neuronal progenitors develop from the ventricular zone in this region at around E10.5–18 [42,47]. Neurogenesis occurs during this interval, ending at around E13 with the differentiation and migration of post-mitotic Purkinje neurons occurring by E13–17 [41,42,47]. Establishment and refinement of input cellular connectivity to Purkinje neurons continue during the postnatal period in mice, extending well past the second postnatal week. Granular neuron precursors cells (GNPCs) migrating from the rhombic lip cover the surface of the developing cerebellum and represent a mitotically active area during postnatal life, with peak proliferation occurring between postnatal days 5 and 8 [41,42]. Extensive proliferation of GNPCs leads to the formation of the external granular layer (EGL) in the cerebellar cortex. GNPC proliferation in the EGL is followed by differentiation and radial migration along projections of Bergman’s glia, leading to formation of the molecular layer and the internal granular layer (IGL) of the cerebellar cortex during the first 2 weeks of postnatal life in mice. Importantly, as noted above, a similar developmental pattern in the human cerebellum occurs between the 24th gestational week and birth. In both mice and humans, the maturation of cellular connectivity in the cerebellar continues long after birth and is not complete in humans until later in childhood. However, species differences in cerebellar development are apparent in that refinement in cellular connectivity begins prenatally in humans, in contrast to the significant refinement of cellular connectivity that occurs postnatally in mice. Finally, because of postnatal development of this region of the brain in mice, many key findings in the current understanding of fundamental aspects of CNS development have been provided by studies of the development of the cerebellum.

In both humans and mice, critical early steps in development are guided by expression of molecular cues that are temporally regulated during CNS development [46]. Similarly, electrical CNS activity increases throughout development and is critical for establishing cellular connectivity required for the optimal function of neural pathways [48]. Thus, there are unique time points and/or intervals during which the developing human CNS could exhibit specific susceptibilities to insults, such as soluble factors that accompany maternal inflammation or intrauterine infections that, in turn, could result in characteristic patterns of altered CNS development without significant cellular death or loss [39]. Modeling the outcomes of MCMV infections in the developing murine CNS therefore offers a valuable experimental system to define the pathways and mechanisms of CNS developmental disruption which follow intrauterine infections, such as those that follow transplacental transmission of HCMV to the developing fetus. 

## 4. Murine Models of CNS Infections Associated with Congenital HCMV Infection: Murine Models of CNS Infection with HCMV

As noted previously, vertical or transplacental transfer of MCMV to the developing fetus does not consistently occur, because MCMV does not efficiently cross the murine placenta. Although both human and murine placentas are classified structurally as hemichorial, the murine placenta consists of additional layers of trophoblasts between the fetal and maternal circulation when compared to the single cell layer in humans, a difference that has been argued to account for the limited transplacental transmission of MCMV [49]. To overcome this difference in placental function in mice, several different strategies have been employed to generate models of embryonic MCMV infection that could, in turn, result in infection of the developing brain. Models include (i) direct injection of the virus either into the placenta or embryo; (ii) direct intracranial inoculation of the virus into the embryo or the newborn pup; and (iii) the intraperitoneal inoculation of newborn pups (Table 3) [50,51,52,53]. Other MCMV infection models have described footpad inoculation, intranasal inoculation, and intravenous tail vein injections of pregnant dams. These latter routes of infection have either not been well studied during neonatal brain development, or have failed to produce similar levels of CNS infections, as reported with infections of embryos or newborn pups.

Several models have been developed to study MCMV infection in the mouse embryo early in development. To circumvent the lack of readily achievable transplacental MCMV transmission, investigators have injected MCMV into the placenta on E12, thus providing entry of MCMV into the fetal circulation early in CNS development. The embryos were allowed to develop in utero, and brains were collected later in gestation (E18), or after delivery, and analyzed. Significant embryo loss occurred after injection; however, infection was achieved in about 30% of surviving embryos [52]. Viral antigen-containing cells were detected in multiple regions of the brain, and in some surviving infected pups, microcephaly was described [52]. This same group of investigators has also described the productive infection of brain-derived neural stem cells and losses of self-renewal, differentiation, and migration of stem cells infected with MCMV [54]. These observations, combined with findings from this model of infection early in embryogenesis, provide evidence that infection of the mouse CNS early in development could lead to significant developmental abnormalities, perhaps secondary to the MCMV infection of neural stem cells during neurogenesis [54]. However, this model system is technically challenging, and because in only about 25% of embryos could infection of the developing brain be demonstrated, this system may be limited in the number of experimental parameters required for investigations of specific mechanisms of MCMV-induced disease. Alternatively, it does provide the capability to achieve infection in early periods of CNS development, an attribute of this model which is particularly invaluable for defining the impact of MCMV infection in early periods of neurogenesis. 

An alternative methodology that has been used to establish infection of the developing CNS is the direct inoculation of MCMV into cerebral ventricles of the embryo [54,55,56,57]. In this approach, mouse embryos are inoculated with MCMV on E15.5 mice by injection through the uterine walls and into the cerebral ventricles [50]. As was observed in the model system in which MCMV was used to infect the placenta of the developing embryo, embryo loss was often significant and unpredictable. Infections of cells expressing neuronal markers were observed in the subventricular zone and in the cortex of surviving embryos [55,63]. Although this provides a predictable CNS infection model in mid-gestational embryos, it circumvents the requirement for hematologic spread to the CNS, a histopathological correlate of neuropathogenesis in the developing human fetus that has been described in autopsied human fetuses infected early in gestation [16]. This potential drawback and the contribution of needle trauma responses in the developing brain that can accompany intracerebral (IC) infection have prompted some caution in the interpretation of data from studies employing this model system. Even with these limitations, this approach does provide an experimental strategy for investigations of the role of MCMV infection in altered morphogenesis and the definition of cell-specific tropism in the developing brain during the mid-period of embryogenesis.

Perhaps the most commonly described model of MCMV infection of the developing brain utilizes direct intracerebral MCMV inoculation into newborn pups within 24 h of birth [64]. This model enables reproducible and quantitative delivery of the virus with high rates of survival of infected mice for studies of infection during postnatal CNS development. Other advantages of this system include the finding that IC inoculations allow the virus to be directly inserted into the brain, thus avoiding the induction of innate and adaptive peripheral immune responses which could dampen or curtail MCMV infection in some mouse strains. This model has been used most extensively to catalog innate and adaptive immune responses in the CNS of young animals and to document persistent infection in the CNS of mice infected as newborn pups [60,61,62]. In addition, this approach has been used by some investigators to induce hearing loss in young mice (see the following sections) [58,59]. As noted above in descriptions of IC injections into the developing embryo, a direct injection of MCMV into the CNS limits any studies on the routes of spread to various regions of the brain and is accompanied by the induction of innate immune responses secondary to tissue injury from the needle track [65]. Perhaps more relevant to understanding the neuropathogenesis of congenital HCMV infections, the lack of the timely induction of peripheral innate and adaptive immune responses in mice inoculated IC could minimize the role of immune responses and inflammation in the pathogenesis of this models of congenital HCMV infection. However, this model should not be summarily dismissed because it does offer distinct advantages for studies of CNS responses, particularly the responses of resident cells such as microglia, that follow CNS infection with a non-neurotropic virus such as CMV. 

A more recent model of congenital HCMV infection was developed following the finding of consistent and predictable patterns of CNS infection following the intraperitoneal (ip) inoculation of newborn mice with relatively small amounts of MCMV. When neonatal mice less than 24 h old are inoculated i.p., MCMV disseminates widely, including to the CNS [53]. In addition, MCMV replicates in the parenchyma of the brain, and a number of different cell types can be shown to be infected, including neuronal cells, astroglial cells, microglial cells, and endothelial cells in the brain vasculature [53,66]. When compared to models of intracranial inoculation, this model provides a clear advantage in that it more closely recapitulates the systemic viremia that occurs in fetuses infected in utero by HCMV, and therefore reflects a physiological mode of virus spread to the CNS. In addition, this mode of spread to the CNS occurs during the induction of both innate and adaptive immune responses in the periphery, although these responses are developmentally immature in newborn mice. As noted above, development of the newborn mice CNS is equivalent to humans at mid-second trimester gestation, a developmental time interval that has been identified as the most frequent time period for HCMV transmission to the fetus [9,11]. Thus, this system recapitulates many key aspects of the vertical transmission of viruses that have been observed in congenital HCMV infection, and importantly, provides an experimentally tractable system to study the impact of virus infection on the developing CNS, particularly the cerebellum. Finally, this model requires minimal technical skill, enables reproducible infection with defined amounts of the virus, and results in the minimal loss of newborn pups when compared to the previously described models. 

## 5. Pathologic and Histopathologic Findings in Murine Models of Congenital HCMV Infections: Clues to Potential Mechanisms of Disease

**Figure 3:**
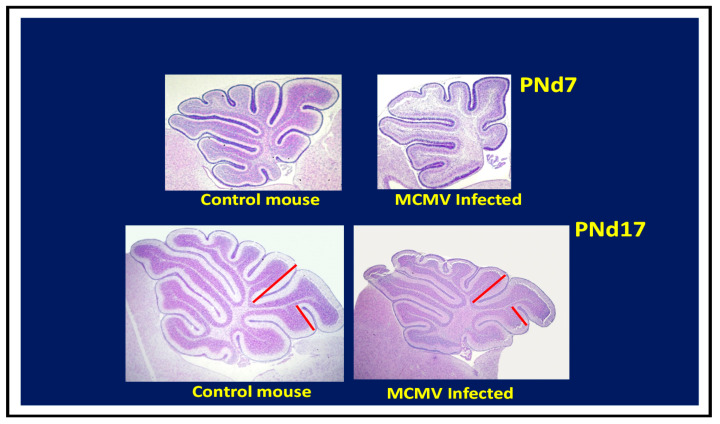
Cerebellar hypoplasia following newborn infection with MCMV. Newborn mice infected intraperitoneally with 200 pfu MCMV or mock infected with media (control) and harvested on postnatal day 7 or 17 (PNd7, 17). Sagittal sections of cerebella stained with cresyl violet demonstrate hypoplastic cerebella from infected mouse with a loss of characteristic foliation of the cortex, including a reduced depth of cortical fissures in both postnatal mice infected on day 7 and 17 compared to control mice (red bars shown for postnatal day 17 mice).

In murine models of congenital HCMV infection that utilize infection early in embryogenesis, such as those that employ placental inoculation or the IC inoculation of embryos, significant structural abnormalities have been described [52]. These include a loss of brain parenchyma with a corresponding loss of cerebrum volume, ventriculomegaly, and microcephaly. Importantly, these findings in murine models are parallel those reported from studies of human fetal tissue derived from terminations of pregnancy that were presumably infected early in gestation [16,17]. Such findings are consistent with decreased neurogenesis, presumably secondary to the loss of neural progenitor cells in the subventricular zone of the developing brain, findings that mirror those of in vitro studies of murine neural stem cells [54]. Similar conclusions have been drawn from studies of human autopsy tissue and also from imaging studies of surviving infected infants (Table 2) [16]. As noted previously, these findings have been associated with a loss of brain parenchyma and were consistent with the clinical findings of microcephaly and ventriculomegaly in infected mouse embryos and newborn pups, as well as in infected human fetuses. Similarly, cerebellar hypoplasia has been described in autopsy tissue from human fetuses infected in utero and from imaging studies of infected infants (see Table 1 and Table 2). Cerebellar hypoplasia is a dominant finding in mice infected as newborns by intraperitoneal inoculation (Figure 3) [53]. In this model, cerebellar hypoplasia and the corresponding decrease in foliation of the adult cerebellum can be attributed to decreased granular cell precursor proliferation [53,67,68]. Together, these data suggest that characteristic pathologic findings described in human autopsy material and imaging studies from infants with congenital HCMV infection can be recapitulated in murine models. 

Histopathological findings in brain tissue from MCMV-infected mice exhibit abnormalities analogous to those described in autopsy tissues from infants infected in utero with HCMV. In the murine models described above, these include the infection of neurons, glial cells and endothelial cells, without evidence of specific cell tropism. There is an absence of widespread cell death or necrosis. In murine models that have utilized either infection of the placenta or direct IC inoculation of the embryo, the loss of brain parenchyma was associated with decreased cerebrum volume and ventriculomegaly. These findings are similar to that reported in autopsy specimens from fetuses infected early in gestation [16,17]. In both mice infected early in development and human fetuses infected at an early gestational age, loss of cortical parenchyma and ventriculomegaly was suggested to follow the loss of neural stem/progenitor cells in the subventricular zone of the developing brain [16,54]. Finally, one of the most common histopathological findings described in both human specimens and in tissue from mouse models is focal micronodular gliosis, a histopathological term which describes a focal accumulation of mononuclear cells surrounding a virus-infected cell. Importantly, there appeared to be no predilection for the distribution of these lesions in specimens from human cases of fetal HCMV infection, and similarly, in the murine model that utilizes intraperitoneal infection of newborn mice [16,53]. These findings imply that virus dissemination in some murine models accurately reflects the hematogenous viral spread to the CNS that follows intrauterine infection of the fetus with HCMV. 

Although the lytic infection of neural progenitor cells early in embryonic development provides a plausible mechanism for the loss of progenitor cells in the subventricular zone and subsequent loss of brain parenchyma seen in both murine models and human fetuses infected at an early gestational age, the loss of cerebellar GNPCs and subsequent symmetric hypoplasia of the cerebellum cannot be explained by a direct effect of a lytic virus infection of the cerebellum. Such findings indicate an indirect effect of virus infection leading to altered neurodevelopment. Mechanisms could include, but are not limited to, disruption of the CNS blood supply and exposure to inflammatory cytokines. Robust inflammatory responses, including large numbers of interferon-inducible genes and TNF-α, are detected in the brains of newborn MCMV-infected mice [53,65,69]. Early cellular immune responses in the first 2–3 days post-infection include the activation of resident microglial cells and recruitment of inflammatory monocytes, neutrophils and natural killer (NK) cells [70]. Shortly thereafter, MCMV-specific CD8+ T cells are recruited; with their appearance, acute infection is resolved, and the infection begins to establish a latent MCMV infection. Thus, the induction of an intense immune response with the resulting release of proinflammatory mediators could also dysregulate critical signaling cascades required for GNPC proliferation and normal neurodevelopment. Several studies have provided data consistent with the role of proinflammatory host responses in altered neurodevelopment in this model, and have identified TNFα and INFγ as key mediators of this immuopathological response [67,68,71].

In summary, there are several of models of MCMV infection that have proven informative in studies of the impact of virus infection on the developing brain, and each model has both specific advantages and limitations. It is important to recognize unique differences between species at specific time points during neurodevelopment and the duration of cellular events that contribute to normal neurodevelopment when attempting to translate findings from murine models to findings in human fetuses and infants with cCMV infections. Intraperitoneal MCMV inoculation of newborn mice best recapitulates hematogenous spread as well as many of the histopathological findings of CNS infection observed in the developing human fetus. Similar histopathological findings are observed (reduced cerebellar foliation, cellular tropism and gliosis, etc.) in infants with cCMV infections and in mice following neonatal MCMV intraperitoneal infection. Finally, in mice and human CMV infections, inductions of immune responses and proinflammatory cytokines both contribute to virus control and to the neuropathology described in CNS infection. Lastly, the method which is selected for infection and the timing of infection are both dependent on the context of the individual study, and more importantly, on the specific goals of the study, because it is unlikely that any single murine model can faithfully recapitulate the complexity of mechanisms of diseases associated with congenital HCMV infection. 

## 6. Hearing Loss and Vestibular Dysfunction Following Congenital HCMV Infections

Sensorineural hearing loss (SNHL) remains the most common sequelae following cCMV infection, with an overall prevalence of between 8% and 12% of all infected infants [1,72]. Hearing loss is more frequently observed in infants with symptomatic infection and is observed in the majority of infants with CNS imaging abnormalities [6,21]. Conversely, a recent study demonstrated that almost all infants who failed newborn hearing screening examinations had CNS imaging findings, suggesting that CNS involvement represented a significant risk factor for hearing loss [73]. In addition, several reports have confirmed results from very early studies which described vestibular dysfunction in infants with cCMV infections [8,74,75,76]. Hearing loss in cCMV-infected infants can range from mild to profound, and curiously, can present with unilateral hearing loss. In addition, delayed onset or progression of hearing loss is not infrequent, particularly in infected infants who initially present with mild hearing loss. As a result, studies that have reported hearing loss without adequate periods of follow-up have often underestimated the rate of hearing loss in infected infants. This is illustrated by the recent report describing the reduced efficacy of postnatal treatment of infants with cCMV infections with the antiviral valganciclovir, which was observed when treated infants were followed for >48 months of life [77].

Comprehensive histopathological studies of the temporal bones of infants with cCMV were very limited until the last decade. In fact, a review of the literature in 2006 noted that histopathology was available on a total of only 12 temporal bones from infants with cCMV infections [78]. Two more recent studies have provided detailed descriptions of the temporal bones from a total of 26 fetuses, all with evidence of disseminated intrauterine cCMV infection, and in most, CNS involvement [79,80]. Evidence of HCMV infection was found throughout the inner ear, including in the stria vascularis, Reissner’s membrane, the spiral ganglion, and the endocochlear duct [79,80]. Importantly, in only one case was there suggestive evidence of infections of hair cells in the sensory epithelium [80]. Extensive infections of structures within the vestibular apparatus were described, including in the dark cells, whereas in the second study, the frequency of infection detected in structures of the vestibular system was significantly less [79,80]. In both studies, there was an infiltrate of mononuclear cells in the inner ear structures that included CD8^+^ T lymphocytes [79,80]. Finally, although these specimens were likely derived from fetuses infected early in gestation, it is important to note that the precise timing of infection of structures in auditory pathways cannot be established with certainty, but only estimated. Most recently, autopsy specimens from two infants with cCMV infection who died during the perinatal period revealed similar findings, except that in one infant, the loss of outer hair cells was reported and infected cells were observed within the cochlear duct, although there was no evidence of virus infection in the hair cells [81]. Histologic changes were noted in the vestibular system with a loss of vestibular hair cells and HCMV inclusions seen in cells adjacent to the dark cells [81]. Together, these studies demonstrate that HCMV can infect a variety of cell types within the inner ear, with the notable exception of hair cells of the Organ of Corti. Furthermore, the presence of both inflammatory infiltrates and the infection of a number of different structures, ranging from the stria vascularis and the spiral ganglion of the cochlea to the dark cells and ganglion cells of the vestibular apparatus, suggest several potential but not mutually exclusive mechanisms of disease. As noted above, the complexity of the biological system and the potential combinatorial nature of the mechanisms of disease, such as the timing of infection and magnitude of inoculum that, in turn, produce the variability in the observed clinical phenotypes, suggest that deciphering these processes will require animal models that faithfully recapitulate the human disease. 

## 7. Murine Models of Hearing Loss Associated with Congenital HCMV Infection

**Figure 4:**
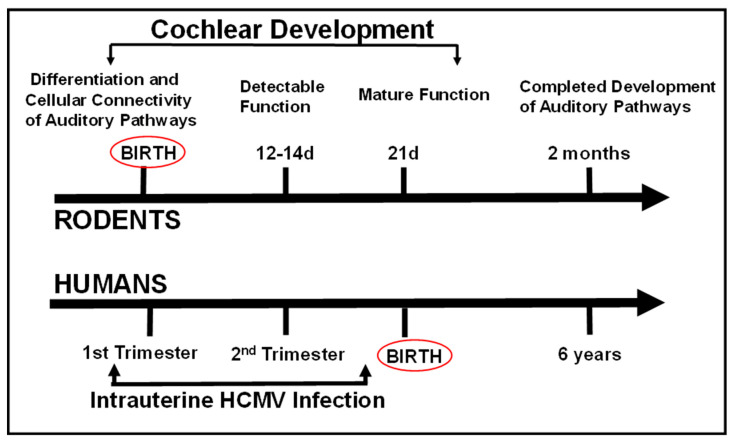
Comparative timelines of auditory development in rodents and in humans. Note that auditory function in human develops during the 2nd trimester of human fetal life, and newborn human infants have well-developed hearing. In contrast, rodents are deaf at birth, and auditory functions mature to adult levels by 3 weeks of age. The timeline of intrauterine human CMV infection for comparison is shown at the bottom of the figure and reflects a similar timeline of infection in the developing rodent auditory system following infections in the newborn period.

The development of auditory pathways in the rodent is parallel to the development of many hindbrain structures in the CNS, a finding which further illustrates that maturation of the many components of the CNS of rodents occurs in the postnatal period (Figure 4). In addition, studies have demonstrated that a significant population of hair cells in the utricle of the inner ear appears during the postnatal period through processes of both proliferation and differentiation of progenitors that have exited the cell-cycle before birth [82]. Thus, a significant portion of auditory development in mice takes place postnatally, providing an ideal experimental system for defining the impact of different insults on the development of the auditory system. As an example, Rubel et al., identified that a critical period during postnatal auditory development existed for establishing connectivity between the sensory epithelium and cochlear nuclei in the brain stem [83]. In this study, the selective destruction of hair cells within the Organ of Corti during early cochlear development resulted in a loss of spiral ganglion neurons as well as neurons in the cochlear nucleus, and interestingly, the progressive loss of neurons in the spiral ganglion [83]. These histopathological changes were associated with profound hearing loss [83]. In contrast, destruction of hair cells in the same experimental system in adult mice, although producing hearing loss, did not result in a loss of neurons in the spiral ganglion or the cochlear nucleus [83]. This series of experiments provided definitive evidence that not only are cellular components of the sensory epithelium essential for the development of hearing, but that normal hearing also requires connectivity and signaling between neural components of the auditory pathway to be established during early development of the auditory system.

Several murine models have been developed to study hearing loss following cCMV infections, and with one exception, several of these models have taken advantage of the finding that a significant degree of auditory development in mice takes place during the postnatal period. The model described by Juanjuan et al. utilized infection of the developing embryo [84]. In this system, placentas in timed pregnancies (E12.5) were injected with MCMV, and embryos were harvested either on E18.5 or allowed to progress to birth [84]. Infection was established in about 30% of embryos, and overall, about 65% of embryos following placental inoculations were live-born [84]. Live-born animals infected as embryos were tested using auditory brainstem responses at PNd28 and PNd78, and at both time points, mice infected as embryos exhibited hearing loss when compared to control animals [84]. Histopathology of the cochlea, combined with DNA in situ hybridization, demonstrated viral DNA in the stria vascularis, the spiral ganglion, and the spiral ligament [84]. Infection or loss of cells of the sensory epithelium, including the supporting cells, was not described. However, there was loss of spiral ganglion neurons [84]. Although this model system has attempted to recapitulate infection early in gestation which is observed in aborted human fetuses and infants with cCMV infection, the system is technically challenging, associated with a significant loss of embryos, and infection could be demonstrated in only one-third of surviving embryos. Furthermore, the histopathology of the infected placentas was not described, and similar to findings in the more well-described guinea pig model of cCMV infections, the contribution of placental damage secondary to MCMV infection and the loss of embryos and outcomes of infected live-born pups, such as hearing loss, remains uncertain [85,86]. However, this system offers a novel approach and remains the only model system that could potentially investigate the parameters of MCMV infection during the very early developmental period of the auditory system and resulting outcomes.

Infections of newborn mice with MCMV represent the most commonly employed model to study mechanisms of hearing loss that follow intrauterine infection with HCMV. Although several models have been described, as a group, these models differ primarily by the route of MCMV infection. As noted in previous sections, in the most commonly used model, newborn mice are inoculated intracerebrally (IC) with purified MCMV. As a result, the virus can replicate in the brain parenchyma and ventricles and spread further to parenchyma of the developing brain (see previous sections), including through the cerebrospinal fluid (CSF) and cochlear aqueduct to the inner ear [87,88]. This route of infection bypasses hematogenous virus spread to the inner ear, the presumed physiological route of infection of the inner ear of infants infected in utero with HCMV, and importantly, also bypasses recognition of the virus by immunological functions present in the periphery [79]. Thus, there are notable differences in the findings in this model when compared to reported findings in the model using peripheral inoculation (see the following section). Perhaps the most obvious differences between the findings in infected mice following IC infection when compared to mice infected by peripheral inoculation is in the histopathology of the inner ear. In most reports utilizing IC inoculations, there is a delayed damage to the sensory epithelium with a significant loss of hair cells [58,59]. Interestingly, IC MCMV infection of newborn mice did not result in the infection of hair cells, but led to MCMV-infected cells in the spiral ganglion, epithelial cells in the scala typmpani and in the stria vascularis, findings that paralleled those in mice infected peripherally [58,59,89]. In addition, the loss of hair cells in the sensory epithelium was noted several weeks following the IC inoculation of newborn mice, suggesting an indirect effect of the sensory epithelium and direct virus-mediated cytopathology in other regions of the inner ear [58,59]. The impact of IC infection on supporting cells of the sensory epithelium was not described in these models, thus raising the possibility that MCMV-induced hair cell loss in these models was secondary to the loss of supporting cell function [90]. Although the loss of hair cells in models using IC inoculation provides an obvious mechanism for the loss of hearing in these animals, loss of the sensory epithelium is not routinely observed in studies of inner ears from fetuses infected in utero with HCMV, nor in mice inoculated peripherally in the newborn period [79,80,91,92]. Thus, the severe damage observed in the sensory epithelium in animals inoculated IC suggests that other, more subtle damage in the cochlea of infected animals could contribute to hearing loss, but such changes could be obscured by overwhelming damage to components of the Organ of Corti. Finally, even though the audiologic findings in mice inoculated IC align with those from human infants with hearing loss associated with cCMV infections, including increased thresholds as measured by otoacoustic emissions and auditory brainstem responses, histological changes in the sensory epithelium of animals inoculated IC have not been consistently reported in histopathological studies of autopsy material from infants with cCMV infections (see the preceding section) [79,80]. Additional histopathologic changes in animals inoculated IC included a loss of spiral ganglion neurons, damage to the stria vascularis, and infiltrations of mononuclear cells of the innate and adaptive immune response [58,59,93]. As noted above, with the exception of damage to the sensory epithelium, similar findings have been reported in newborn mice infected with MCMV by peripheral inoculation [91,92]. However, it should be noted that the direct IC inoculation of newborn animals does offer an important advantage in that severe hearing loss occurs in a significant proportion of animals, and secondly, provides an experimental approach to aid in defining genetic determinants of viral virulence such as neuroinvasion and immune evasion.

Initial efforts using routes of peripheral infections such as intraperitoneal (IP) or intravenous (IV) injections to infect the inner ear of mice often failed or were highly inefficient. One group of investigators employed an IP route of infection in two-day-old animals and facilitated spread to the CNS by the IC inoculation of lipopolysaccharide (LPS) to establish an inflammatory response, and presumably, increase permeability of the vasculature in the CNS and inner ear by disruption of the blood/labyrinth barrier (BLB) [94]. This approach led to infection of the inner ear with the detection of MCMV in most compartments, with the notable exception of the Organ of Corti [94]. In addition, this approach led to significantly increased hearing thresholds in animals administered MCMV and LPS [94]. Other routes, including trans-tympanic membrane inoculations, have also been employed, but again failed to induce hearing loss in a significant proportion of infected animals [59]. However, in the last decade, several groups of investigators have demonstrated that the IP infection of newborn mice shortly after birth (< 24 h of age) can result in peripheral virus replication, viremia, and hematogenous dissemination to the brain and inner ear (see preceding sections) [53,91]. This model system has provided a convenient and informative animal model that faithfully recapitulates many of the characteristics of hearing loss that follow cCMV infections, including: (i) variability in the magnitude of hearing loss, ranging from mild to severe; (ii) delayed onset of hearing loss in some animals; (iii) unilateral hearing loss in some animals; and (iv) progressive hearing loss [91]. Infection of newborn mice with relatively small amounts of tissue culture passaged wild-type Smith strain MCMV resulted in a significant number of animals that developed hearing loss, and importantly, limited mortality, thereby providing a sufficient number of infected animals that could be subjected to repeated audiologic testing after maturation of their hearing. In contrast to some reported models utilizing IC inoculations in which the death of mice infected as newborns was nearly universal by postnatal day 30 (PNd30), this model system provides the opportunity to test animals later in life for the delayed onset of hearing loss and progressive hearing loss [91]. Perhaps the most important attribute of this model is that IP inoculation results in the hematogenous spread of the virus to the inner ear, thus recapitulating the route of virus spread to the inner ear of the fetus that occurs in human intrauterine infections [79]. Finally, systemic immune responses comprising both innate and adaptive effector functions develop in response to dissemination of the virus infection, and these responses appear to control virus replication in the inner ear. These same responses also result in a robust inflammatory response that are thought to contribute to abnormalities in the development of auditory system that lead to hearing loss in infected animals [91,92].

**Figure 5:**
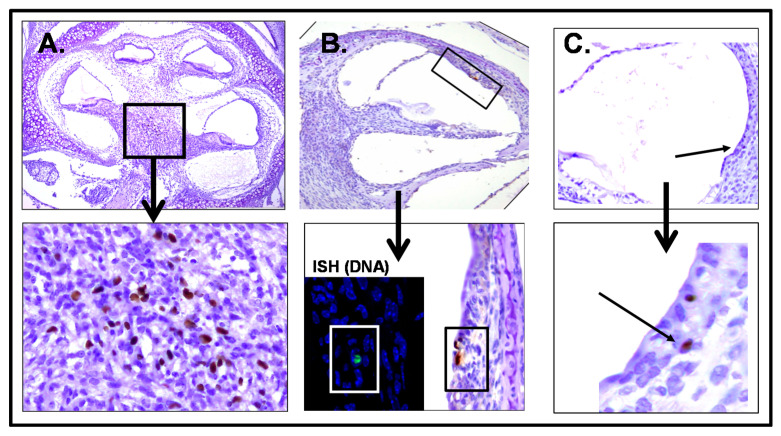
MCMV infection of the cochlea of mice infected as newborns. MCMV infects cells of cochlea in neonatal mice. Following perfusion, cochlea were removed, fixed, paraffin-embedded and prepared for immunohistochemistry using anti-MCMV antibody (Croma1) and developed with DAB (brown). Boxed area has been digitally enlarged (arrow). (**A**) Spiral ganglion postnatal day 4 (PNd4); (**B**,**C**) stria vascularis postnatal day 11 (PNd11). Note: infected cells are illustrated by arrows in panel (**C**). The inset in lower panel B shows viral DNA + cell (green) detected by in situ DNA hybridization (ISH) in tissue adjacent to a section containing the MCMV-infected cell.

Histologic findings in sections from the inner ear of mice infected with MCMV in the newborn period revealed virus replication in a number of sites in the cochlea of infected animals (Figure 5). These include both the spiral ganglion and the stria vascularis, similar to the distribution in models utilizing IC inoculations, and importantly, in findings from autopsy specimens of fetuses infected in utero (Figure 5) [16,17,58,59].

**Figure 6:**
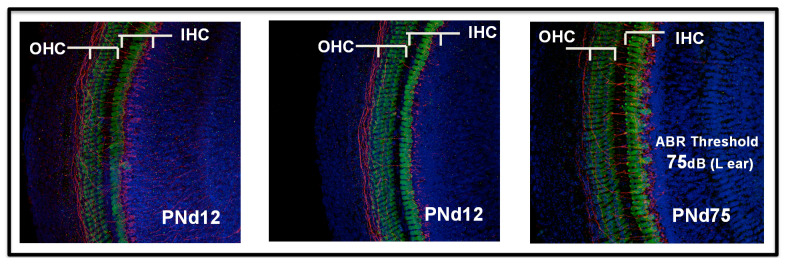
Preservation of the sensory epithelium in the Organ of Corti from mice infected as newborns. Newborn Balb/c mice were infected with 200 pfu MCMV on postnatal day 0 (PNd0) and harvested on postnatal day 12 (PNd12) or postnatal day 75 (PNd75), as indicated. Following cochlear perfusion and whole mount preparation, hair cells were stained with anti-myosin 7 and developed with FITC conjugated secondary antibody (green). Neurofilaments were stained with anti-NF antibodies and developed with alexafluor594 conjugated secondary antibody (red). Nuclei were stained with DAPI (blue). Inner (IHC) and outer (OHC) cells are indicated. Note that panel C is from a mouse with severe hearing loss.

**Figure 7:**
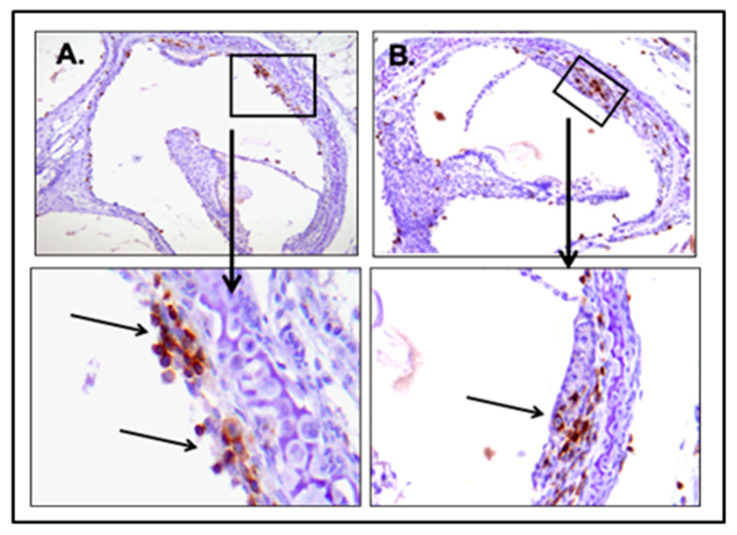
CD3^+^ T lymphocytes infiltrate the cochlea in mice infected with MCMV. Newborn Balb/c mice were infected intraperitoneally with 200 pfu and harvested on postnatal day 12 (PNd12). Following fixation and sectioning, sections from the cochlea were stained with anti-CD3^+^ antibodies and developed with histochemical staining. Brown signals indicate CD3^+^ T lymphocytes, including in the wall of the vestibular canal (Panel **A**) and in the stria vascularis (Panel **B**).

As in other models, MCMV was not detected in the sensory epithelium in newborn mice infected by the IP route, and the sensory epithelium remained intact in animals infected by an IP inoculation, although findings consistent with an auditory neuropathy were present (Figure 6) [91,92]. A mononuclear infiltrate was observed in the cochlea of mice infected as newborns by IP injection, including both activated monocytes and CD3^+^ T lymphocytes (Figure 7). A robust induction of proinflammatory molecules, including interferon-stimulated genes, was associated with the infiltration of mononuclear into the cochlea [91,92]. Finally, abnormal hearing in infected mice, as measured with both DPOAE and ABR testing, was demonstrated in about 30–50% of animals [91,92]. The magnitude of hearing loss was variable, ranging from 10 dB changes in thresholds to >100 dB, and as noted above, delayed onset, progressive, and unilateral hearing loss could observed in this model [91,92].

## 8. Summary

Intrauterine HCMV infections of the developing fetus and the resulting adverse outcomes in infants with cCMV infection are of considerable importance because of the relatively common occurrence of this perinatal infection worldwide. Although investigators have long recognized the spectrum of clinical findings in infants with cCMV, more recent studies have strongly argued that the characteristics of CNS involvement with this infection are primary determinants of the long-term outcomes of infected infants. Similar to the challenges presented by investigations of other diseases of the human CNS, informative model systems are absolutely essential for defining mechanisms of disease in infants with cCMV infections. Although studies in CNS organoids offer exciting new opportunities for mechanistic studies that can be further refined in vitro, animal models remain the mainstay for investigating the impact of HCMV infection on the developing CNS. Murine models of CNS infection have been the most well characterized and offer numerous advantages over both nonhuman primates and other small-animal models secondary to the extensive amount of existing information on neurodevelopment and immunological development of laboratory mice. Currently employed models have taken advantage of the well-described CNS development in mice and have modeled the impact of virus infection at various time points during CNS development, including the development of auditory pathways in postnatal mice. These models have provided new understandings of mechanisms of virus-induced neurodevelopmental disease, including hearing loss. Findings from these murine models parallel histopathological findings in tissues from HCMV-infected fetuses, and importantly, in some cases, closely recapitulate clinical phenotypes of infants with cCMV infections. Further refinement of these models can be expected to enable more directed studies of mechanisms of disease that lead to adverse outcomes in infants with cCMV infections, and almost assuredly, the design and testing of interventions to modify long-term outcomes of infants infected in utero with HCMV.

## Figures and Tables

**Table 1:** Clinical findings in infants with symptomatic congenital CMV infections.

Non-Central Nervous System Findings	Central Nervous System Findings
Jaundice	Microcephaly
Hepatosplenomegaly	Seizures
Purpura (thrombocytopenia)	Chorioretinitis
Intrauterine Growth Restriction	Abnormal neurologic findings(seizures, motor and cognitive deficits; delays in neurodevelopment)
	Hearing loss (failed hearing screening)
	Vestibular disturbances

**Table 3:** Murine models of CNS infection following intrauterine human cytomegalovirus infection.

Model ^1^	Description	Advantages	Limitations
Placental Injection [52,54]	Placental inoculation E12	- Embryo infection early in CNS development.	- Significant embryo loss; - Technically difficult.
Embryo Infection [55,56,57,58,59]	IC injection E15	- Embryo infection early in CNS development; - Predictable CNS infection.	- Non-physiologic CNS infection; - CNS needle trauma; - Technically difficult.
IC inoculation newborn pup [60,61,62]	IC injection <24 h of age	- Quantitative virus delivery; - High survival; - Limited peripheral immune response early after infection.	- Non-physiologic CNS infection; - CNS needle trauma.
IP inoculation newborn pup [53]	IP injection <24 h of age	- Quantitative virus delivery; - High survival; - Physiologic virus spread to CNS; - Peripheral immune response to infection; - CNS development similar to 2nd trimester fetus	- Cannot model infection during early cortical development.

^1^ Experimental models utilized intraplacental inoculations, intracerebral inoculations (ICs), and intraperitoneal inoculations (IP). References describing these models are indicated.

## Abbreviations

BLB	blood/labyrinth barrier
cCMV	congenital cytomegalovirus
CNS	central nervous system
CT	computerized tomography
EGL	external granular layer
GNPC	granular neuron precursor cell
HCMV	human cytomegalovirus
HSV	herpes simplex virus
IC	intracerebral
IP	intraperitoneal
MCMV	murine cytomegalovirus
MRI	magnetic resonance imaging
SNHL	sensorineural hearing loss
SVZ	subventricular zone
US	ultrasound

## References

[1] Fowler K.B., Boppana S.B. (2018). Congenital cytomegalovirus infection. Semin Perinatol..

[2] Manicklal S., Emery V.C., Lazzarotto T., Boppana S.B., Gupta R.K. (2013). The “silent“ global burden of congenital cytomegalovirus. Clin. Microbiol. Rev..

[3] Kenneson A., Cannon M.J. (2007). Review and meta-analysis of the epidemiology of congenital cytomegalovirus (CMV) infection. Rev. Med. Virol..

[4] Stagno S., Pass R.F., Dworsky M.E., Alford C.A. (1983). Congenital and perinatal cytomegaloviral infections. Semin. Perinatol..

[5] Boppana S.B., Pass R.F., Britt W.J., Stagno S., Alford C.A. (1992). Symptomatic congenital cytomegalovirus infection: Neonatal morbidity and mortality. Pediatr. Infect. Dis. J..

[6] Dreher A.M., Arora N., Fowler K.B., Novak Z., Britt W.J., Boppana S.B., Ross S.A. (2014). Spectrum of disease and outcome in children with symptomatic congenital cytomegalovirus infection. J. Pediatr..

[7] Dhondt C., Maes L., Rombaut L., Martens S., Vanaudenaerde S., Van Hoecke H., De Leenheer E., Dhooge I. (2020). Vestibular function in children with a congenital cytomegalovirus infection: 3 years of follow-up. Ear Hear..

[8] Pinninti S., Christy J., Almutairi A., Cochrane G., Fowler K.B., Boppana S. (2021). Vestibular, gaze, and balance disorders in asymptomatic congenital cytomegalovirus infection. Pediatrics.

[9] Stagno S., Pass R.F., Cloud G., Britt W.J., Henderson R.E., Walton P.D., Veren D.A., Page F., Alford C.A. (1986). Primary cytomegalovirus infection in pregnancy. Incidence, transmission to fetus, and clinical outcome. JAMA.

[10] Pass R.F., Fowler K.B., Boppana S.B., Britt W.J., Stagno S. (2006). Congenital cytomegalovirus infection following first trimester maternal infection: Symptoms at birth and outcome. J. Clin. Virol..

[11] Enders G., Daiminger A., Bader U., Exler S., Enders M. (2011). Intrauterine transmission and clinical outcome of 248 pregnancies with primary cytomegalovirus infection in relation to gestational age. J. Clin. Virol..

[12] Faure-Bardon V., Magny J.F., Parodi M., Couderc S., Garcia P., Maillotte A.M., Benard M., Pinquier D., Astruc D., Patural H. (2019). Sequelae of congenital cytomegalovirus following maternal primary infections are limited to those acquired in the first trimester of pregnancy. Clin. Infect. Dis..

[13] Boppana S.B., Ross S.A., Fowler K.B. (2013). Congenital cytomegalovirus infection: Clinical outcome. Clin. Infect. Dis..

[14] Becroft D.M.O., Rosenberg H.S., Bernstein J. (1981). Prenatal cytomegalovirus infection: Epidemiology, pathology, and pathogenesis. Perspective in Pediatric Pathology.

[15] Arribas J.R., Storch G.A., Clifford D.B., Tselis A.C. (1996). Cytomegalovirus encephalitis. Ann. Intern. Med..

[16] Teissier N., Fallet-Bianco C., Delezoide A.L., Laquerrière A., Marcorelles P., Khung-Savatovsky S., Nardelli J., Cipriani S., Csaba Z., Picone O. (2014). Cytomegalovirus-Induced brain malformations in fetuses. J. Neuropathol. Exp. Neurol..

[17] Gabrielli L., Bonasoni M.P., Santini D., Piccirilli G., Chiereghin A., Petrisli E., Dolcetti R., Guerra B., Piccioli M., Lanari M. (2012). Congenital cytomegalovirus infection: Patterns of fetal brain damage. Clin. Microbiol. Infect..

[18] Sellier Y., Marliot F., Bessières B., Stirnemann J., Encha-Razavi F., Guilleminot T., Haicheur N., Pages F., Ville Y., Leruez-Ville M. (2020). Adaptive and Innate immune cells in fetal human cytomegalovirus-infected brains. Microorganisms.

[19] Hayward J.C., Titelbaum D.S., Clancy R.R., Zimmerman R.A. (1991). Lissencephaly-Pachygyria associated with congenital cytomegalovirus infection. J. Child Neurol..

[20] Bale J.F., Bray P.F., Bell W.E. (1985). Neuroradiographic abnormalities in congenital cytomegalovirus infection. Pediatr. Neurol..

[21] Boppana S.B., Fowler K.B., Vaid Y., Hedlund G., Stagno S., Britt W.J., Pass R.F. (1997). Neuroradiographic findings in the newborn period and long-term outcome in children with symptomatic congenital cytomegalovirus infection. Pediatrics.

[22] Barkovich A.J., Lindan C.E. (1994). Congenital cytomegalovirus infection of the brain: Imaging analysis and embryologic considerations. Am. J. Neuroradiol..

[23] Sison S.L., O’Brien B.S., Johnson A.J., Seminary E.R., Terhune S.S., Ebert A.D. (2019). Human cytomegalovirus disruption of calcium signaling in neural progenitor cells and organoids. J. Virol..

[24] Brown R.M., Rana P., Jaeger H.K., O’Dowd J.M., Balemba O.B., Fortunato E.A. (2019). Human cytomegalovirus compromises development of cerebral organoids. J. Virol..

[25] Rolland M., Li X., Sellier Y., Martin H., Perez-Berezo T., Rauwel B., Benchoua A., Bessières B., Aziza J., Cenac N. (2016). PPARγ is activated during congenital cytomegalovirus infection and inhibits neuronogenesis from human neural stem cells. PLoS Pathog..

[26] Rolland M., Martin H., Bergamelli M., Sellier Y., Bessières B., Aziza J., Benchoua A., Leruez-Ville M., Gonzalez-Dunia D., Chavanas S. (2021). Human cytomegalovirus infection is associated with increased expression of the lissencephaly gene PAFAH1B1 encoding LIS1 in neural stem cells and congenitally infected brains. J. Pathol..

[27] Luo M.H., Hannemann H., Kulkarni A.S., Schwartz P.H., O’Dowd J.M., Fortunato E.A. (2010). Human cytomegalovirus infection causes premature and abnormal differentiation of human neural progenitor cells. J. Virol..

[28] Odeberg J., Wolmer N., Falci S., Westgren M., Seiger A., Söderberg-Nauclér C. (2006). Human cytomegalovirus inhibits neuronal differentiation and induces apoptosis in human neural precursor cells. J. Virol..

[29] Lazar A., Löfkvist U., Verrecchia L., Karltorp E. (2021). Identical twins affected by congenital cytomegalovirus infections showed different audio-vestibular profiles. Acta Paediatrica.

[30] Ouellette C.P., Sánchez P.J., Xu Z., Blankenship D., Zeray F., Ronchi A., Shimamura M., Chaussabel D., Lee L., Owen K.E. (2020). Blood genome expression profiles in infants with congenital cytomegalovirus infection. Nat. Commun..

[31] Pinninti S.G., Rodgers M.D., Novak Z., Britt W.J., Fowler K.B., Boppana S.B., Ross S.A. (2016). Clinical Predictors of Sensorineural Hearing Loss and Cognitive Outcome in Infants with Symptomatic Congenital Cytomegalovirus Infection. Pediatr. Infect. Dis. J..

[32] Benoist G., Salomon L.J., Mohlo M., Suarez B., Jacquemard F., Ville Y. (2008). Cytomegalovirus-related fetal brain lesions: Comparison between targeted ultrasound examination and magnetic resonance imaging. Ultrasound Obstet. Gynecol..

[33] Doneda C., Parazzini C., Righini A., Rustico M., Tassis B., Fabbri E., Arrigoni F., Consonni D., Triulzi F. (2010). Early cerebral lesions in cytomegalovirus infection: Prenatal MR imaging. Radiology.

[34] Diogo M.C., Glatter S., Binder J., Kiss H., Prayer D. (2020). The MRI spectrum of congenital cytomegalovirus infection. Prenat. Diagn..

[35] White A.L., Hedlund G.L., Bale J.F. (2014). Congenital cytomegalovirus infection and brain clefting. Pediatr. Neurol..

[36] De Vries L.S., Verboon-Maciolek M.A., Cowan F.M., Groenendaal F. (2006). The role of cranial ultrasound and magnetic resonance imaging in the diagnosis of infections of the central nervous system. Early Hum. Dev..

[37] Sugita K., Ando M., Makino M., Takanashi J., Fujimoto N., Niimi H. (1991). Magnetic resonance imaging of the brain in congenital rubella virus and cytomegalovirus infections. Neuroradiology.

[38] Clancy B., Darlington R.B., Finlay B.L. (2001). Translating developmental time across mammalian species. Neuroscience.

[39] Schepanski S., Buss C., Hanganu-Opatz I.L., Arck P.C. (2018). Prenatal Immune and endocrine modulators of offspring’s brain development and cognitive functions later in life. Front. Immunol..

[40] Chini M., Pöpplau J.A., Lindemann C., Carol-Perdiguer L., Hnida M., Oberländer V., Xu X., Ahlbeck J., Bitzenhofer S.H., Mulert C. (2020). Resolving and Rescuing Developmental Miswiring in a Mouse Model of Cognitive Impairment. Neuron.

[41] Goldowitz D., Hamre K. (1998). The cells and molecules that make a cerebellum. Trends Neurosci..

[42] Hatten M.E. (1993). The role of migration in central nervous system neuronal development. Curr. Opin. Neurobiol..

[43] Leto K., Arancillo M., Becker E.B., Buffo A., Chiang C., Ding B., Dobyns W.B., Dusart I., Haldipur P., Hatten M.E. (2016). Consensus paper: Cerebellar development. Cerebellum.

[44] Haldipur P., Aldinger K.A., Bernardo S., Deng M., Timms A.E., Overman L.M., Winter C., Lisgo S.N., Razavi F., Silvestri E. (2019). Spatiotemporal expansion of primary progenitor zones in the developing human cerebellum. Science.

[45] Abrahám H., Tornóczky T., Kosztolányi G., Seress L. (2001). Cell formation in the cortical layers of the developing human cerebellum. Int. J. Dev. Neurosci..

[46] Sereno M.I., Diedrichsen J., Tachrount M., Testa-Silva G., d’Arceuil H., De Zeeuw C. (2020). The human cerebellum has almost 80% of the surface area of the neocortex. Proc. Natl. Acad. Sci. USA.

[47] Butts T., Green M.J., Wingate R.J. (2014). Development of the cerebellum: Simple steps to make a ‘little brain’. Development.

[48] Chini M., Hanganu-Opatz I.L. (2021). Prefrontal cortex development in health and disease: Lessons from rodents and humans. Trends Neurosci..

[49] Johnson K.P. (1969). Mouse cytomegalovirus: Placental infection. J. Infect. Dis..

[50] Shinmura Y., Aiba-Masago S., Kosugi I., Li R.Y., Baba S., Tsutsui Y. (1997). Differential expression of the immediate-early and early antigens in neuronal and glial cells of developing mouse brains infected with murine cytomegalovirus. Am. J. Pathol..

[51] Kosugi I., Arai Y., Baba S., Kawasaki H., Iwashita T., Tsutsui Y. (2021). Prolonged activation of cytomegalovirus early gene e1-promoter exclusively in neurons during infection of the developing cerebrum. Acta Neuropathologica Commun..

[52] Li R.Y., Tsutsui Y. (2000). Growth retardation and microcephaly induced in mice by placental infection with murine cytomegalovirus. Teratology.

[53] Koontz T., Bralic M., Tomac J., Pernjak-Pugel E., Bantug G., Jonjic S., Britt W.J. (2008). Altered development of the brain after focal herpesvirus infection of the central nervous system. J. Exp. Med..

[54] Kosugi I., Shinmura Y., Kawasaki H., Arai Y., Li R.Y., Baba S., Tsutsui Y. (2000). Cytomegalovirus infection of the central nervous system stem cells from mouse embryo: A model for developmental brain disorders induced by cytomegalovirus. Lab. Investig..

[55] Naruse I., Tsutsui Y. (1989). Brain abnormalities induced by murine cytomegalovirus injected into the cerebral ventricles of mouse embryos exo utero. Teratology.

[56] Tsutsui Y., Kashiwai A., Kawamura N., Aiba-Masago S., Kosugi I. (1995). Prolonged infection of mouse brain neurons with murine cytomegalovirus after pre- and perinatal infection. Arch. Virol..

[57] Shinmura Y., Kosugi I., Aiba-Masago S., Baba S., Yong L.R., Tsutsui Y. (1997). Disordered migration and loss of virus-infected neuronal cells in developing mouse brains infected with murine cytomegalovirus. Acta Neuropathologica.

[58] Schachtele S.J., Mutnal M.B., Schleiss M.R., Lokensgard J.R. (2011). Cytomegalovirus-Induced sensorineural hearing loss with persistent cochlear inflammation in neonatal mice. J. Neurovirol..

[59] Wang Y., Patel R., Ren C., Taggart M.G., Firpo M.A., Schleiss M.R., Park A.H. (2013). A comparison of different murine models for cytomegalovirus-induced sensorineural hearing loss. Laryngoscope.

[60] Mutnal M.B., Hu S., Lokensgard J.R. (2012). Persistent humoral immune responses in the CNS limit recovery of reactivated murine cytomegalovirus. PLoS ONE.

[61] Prasad S., Hu S., Sheng W.S., Singh A., Lokensgard J.R. (2015). Tregs modulate lymphocyte proliferation, activation, and resident-memory T-cell accumulation within the brain during MCMV infection. PLoS ONE.

[62] Cheeran M.C., Gekker G., Hu S., Palmquist J.M., Lokensgard J.R. (2005). T cell-mediated restriction of intracerebral murine cytomegalovirus infection displays dependence upon perforin but not interferon-gamma. J. Neurovirol..

[63] Tsutsui Y., Kosugi I., Kawasaki H. (2005). Neuropathogenesis in cytomegalovirus infection: Indication of the mechanisms using mouse models. Rev. Med. Virol..

[64] Tsutsui Y., Kawasaki H., Kosugi I. (2002). Reactivation of latent cytomegalovirus infection in mouse brain cells detected after transfer to brain slice cultures. J. Virol..

[65] Van den Pol A.N., Robek M.D., Ghosh P.K., Ozduman K., Bandi P., Whim M.D., Wollmann G. (2007). Cytomegalovirus induces interferon-stimulated gene expression and is attenuated by interferon in the developing brain. J. Virol..

[66] Bantug G.R., Cekinovic D., Bradford R., Koontz T., Jonjic S., Britt W.J. (2008). CD8^+^ T lymphocytes control murine cytomegalovirus replication in the central nervous system of newborn animals. J. Immunol..

[67] Kosmac K., Bantug G.R., Pugel E.P., Cekinovic D., Jonjic S., Britt W.J. (2013). Glucocorticoid treatment of MCMV infected newborn mice attenuates CNS inflammation and limits deficits in cerebellar development. PLoS Pathog..

[68] Kveštak D., Juranić Lisnić V., Lisnić B., Tomac J., Golemac M., Brizić I., Indenbirken D., Cokarić Brdovčak M., Bernardini G., Krstanović F. (2021). NK/ILC1 cells mediate neuroinflammation and brain pathology following congenital CMV infection. J. Exp. Med..

[69] Cheeran M.C., Hu S., Yager S.L., Gekker G., Peterson P.K., Lokensgard J.R. (2001). Cytomegalovirus induces cytokine and chemokine production differentially in microglia and astrocytes: Antiviral implications. J. Neurovirol..

[70] Kosugi I., Kawasaki H., Arai Y., Tsutsui Y. (2002). Innate immune responses to cytomegalovirus infection in the developing mouse brain and their evasion by virus-infected neurons. Am. J. Pathol..

[71] Seleme M.C., Kosmac K., Jonjic S., Britt W.J. (2017). Tumor necrosis factor alpha-induced recruitment of inflammatory mononuclear cells leads to inflammation and altered brain development in murine cytomegalovirus-infected newborn mice. J. Virol..

[72] Yamamoto A.Y., Anastasio A.R.T., Massuda E.T., Isaac M.L., Manfredi A.K.S., Cavalcante J.M.S., Carnevale-Silva A., Fowler K.B., Boppana S., Britt W.J. (2019). Contribution of congenital cytomegalovirus (cCMV) to permanent hearing loss in a highly seropositive population: “The BraCHS study“. Clin. Infect. Dis..

[73] Hranilovich J.A., Park A.H., Knackstedt E.D., Ostrander B.E., Hedlund G.L., Shi K., Bale J.F. (2020). Brain magnetic resonance imaging in congenital cytomegalovirus with failed newborn hearing screen. Pediatr. Neurol..

[74] Bernard S., Wiener-Vacher S., Van Den Abbeele T., Teissier N. (2015). Vestibular disorders in children with congenital cytomegalovirus infection. Pediatrics.

[75] Zagólski O. (2008). Vestibular-Evoked myogenic potentials and caloric stimulation in infants with congenital cytomegalovirus infection. J. Laryngol. Otol..

[76] Karltorp E., Löfkvist U., Lewensohn-Fuchs I., Lindström K., Westblad M.E., Fahnehjelm K.T., Verrecchia L., Engman M.L. (2014). Impaired balance and neurodevelopmental disabilities among children with congenital cytomegalovirus infection. Acta Paediatrica.

[77] McCrary H., Sheng X., Greene T., Park A. (2019). Long-term hearing outcomes of children with symptomatic congenital CMV treated with valganciclovir. Int. J. Pediatr. Otorhinolaryngol..

[78] Boppana S., Britt W., Newton V.E., Vallely P.J. (2006). Cytomegalovirus. Infection and Hearing Impairment.

[79] Teissier N., Bernard S., Quesnel S., Van Den Abbeele T. (2016). Audiovestibular consequences of congenital cytomegalovirus infection. Eur. Ann. Otorhinolaryngol. Head Neck Dis..

[80] Gabrielli L., Bonasoni M.P., Santini D., Piccirilli G., Chiereghin A., Guerra B., Landini M.P., Capretti M.G., Lanari M., Lazzarotto T. (2013). Human fetal inner ear involvement in congenital cytomegalovirus infection. Acta Neuropathologica Commun..

[81] Tsuprun V., Keskin N., Schleiss M.R., Schachern P., Cureoglu S. (2019). Cytomegalovirus-induced pathology in human temporal bones with congenital and acquired infection. Am. J. Otolaryngol..

[82] Burns J.C., On D., Baker W., Collado M.S., Corwin J.T. (2012). Over half the hair cells in the mouse utricle first appear after birth, with significant numbers originating from early postnatal mitotic production in peripheral and striolar growth zones. J. Assoc. Res. Otolaryngol..

[83] Tong L., Strong M.K., Kaur T., Juiz J.M., Oesterle E.C., Hume C., Warchol M.E., Palmiter R.D., Rubel E.W. (2015). Selective deletion of cochlear hair cells causes rapid age-dependent changes in spiral ganglion and cochlear nucleus neurons. J. Neurosci..

[84] Juanjuan C., Yan F., Li C., Haizhi L., Ling W., Xinrong W., Juan X., Tao L., Zongzhi Y., Suhua C. (2011). Murine model for congenital CMV infection and hearing impairment. Virol. J..

[85] Park A.H., Gifford T., Schleiss M.R., Dahlstrom L., Chase S., McGill L., Li W., Alder S.C. (2010). Development of cytomegalovirus-mediated sensorineural hearing loss in a Guinea pig model. Arch. Otolaryngol. Head Neck Surg..

[86] Roark H.K., Jenks J.A., Permar S.R., Schleiss M.R. (2020). Animal models of congenital cytomegalovirus transmission: Implications for vaccine development. J. Infect. Dis..

[87] Salt A.N., Plontke S.K. (2018). Pharmacokinetic principles in the inner ear: Influence of drug properties on intratympanic applications. Hear. Res..

[88] Stöver T., Yagi M., Raphael Y. (2000). Transduction of the contralateral ear after adenovirus-mediated cochlear gene transfer. Gene Ther..

[89] Davis G.L., Hawrisiak M.M. (1977). Experimental cytomegalovirus infection and the developing mouse inner ear: In vivo and in vitro studies. Lab. Investig. J. Tech. Methods Pathol..

[90] Wan G., Corfas G., Stone J.S. (2013). Inner ear supporting cells: Rethinking the silent majority. Semin. Cell Dev. Biol..

[91] Bradford R.D., Yoo Y.G., Golemac M., Pugel E.P., Jonjic S., Britt W.J. (2015). Murine CMV-induced hearing loss is associated with inner ear inflammation and loss of spiral ganglia neurons. PLoS Pathog..

[92] Sung C.Y.W., Seleme M.C., Payne S., Jonjic S., Hirose K., Britt W. (2019). Virus-Induced cochlear inflammation in newborn mice alters auditory function. JCI Insight.

[93] Carraro M., Almishaal A., Hillas E., Firpo M., Park A., Harrison R.V. (2017). Cytomegalovirus (CMV) infection causes degeneration of cochlear vasculature and hearing loss in a mouse model. J. Assoc. Res. Otolaryngol..

[94] Li L., Kosugi I., Han G.P., Kawasaki H., Arai Y., Takeshita T., Tsutsui Y. (2008). Induction of cytomegalovirus-infected labyrinthitis in newborn mice by lipopolysaccharide: A model for hearing loss in congenital CMV infection. Lab. Investig..

